# A Systems‐Based Approach for Analyzing Interorganizational Coordination Within Port Risk Control Structures

**DOI:** 10.1111/risa.70325

**Published:** 2026-08-09

**Authors:** Elvira Meléndez, Floris Goerlandt

**Affiliations:** ^1^ Department of Industrial Engineering Dalhousie University Halifax Nova Scotia Canada

**Keywords:** coordination mechanisms, interorganizational risk management, PRCS (Port Risk Control Structure), STAMP (Systems‐Theoretic Accident Model and Processes), systemic risk

## Abstract

This study proposes a systems‐based analysis approach for analyzing interorganizational coordination in complex sociotechnical systems through the interorganizational risk management coordination taxonomy (IRM‐CT). Unlike traditional risk‐analysis approaches that primarily focus on intraorganizational controls, probabilistic assessment, or component reliability, the IRM‐CT operationalizes interorganizational coordination as a systemic control problem embedded within distributed governance and feedback structures. Building on the STAMP‐based Port Risk Control Structure (PRCS), the IRM‐CT distinguishes between operational adaptors, which enact coordination in practice, and enabling adaptors, which provide the structural, procedural, and relational conditions that sustain coordination across organizational boundaries. The approach integrates coordination theory, sociotechnical systems thinking, and resilience engineering, embedding coordination within control and feedback loops and evaluating adaptor configurations through three system value conditions: accountability, predictability, and common understanding. A proof‐of‐concept analysis using the Halifax Port Authority Port Information Guide demonstrates how the IRM‐CT identifies adaptor configurations, diagnoses “*Supported*,” “*Fragile*,” and “*Orphan*” coordination states, and evaluates their contribution to accountability, predictability, and common understanding. The IRM‐CT advances systemic risk governance by revealing how authority, codification, and trust interact to sustain safe control. Beyond ports, the proposed approach may be applicable to high‐risk sectors such as mining, aviation, and energy, offering a structured means for diagnosing and strengthening interorganizational coordination.

## 1 Introduction

Modern port operations involve the joint participation of multiple independent organizations, including port authorities, terminal operators, customs agencies, shipping lines, and regulatory bodies. These actors collectively manage safety‐ and risk‐critical activities such as vessel traffic control, cargo handling, bunkering, and emergency response. These operations occur in an environment that is highly regulated, technologically dynamic, globally interconnected and increasingly subject to digitalization, automation, and the demands of sustainability (Geerlings et al. 2018; Meléndez and Goerlandt 2025a). Despite significant investments in formal risk and safety management systems, port risk governance remains challenged by the fragmentation of control structures and the diffusion of responsibility across organizational boundaries. Such fragmentation compromises not only operational efficiency but also systemic risk governance, as misaligned roles and ineffective feedback loops allow risks to propagate across organizations and networked systems (Nikghadam et al. 2022; Zhang et al. 2024).

Within the risk‐analysis literature, the concept of systemic risk has emerged as a central concern for complex, interconnected systems where small perturbations can escalate into large‐scale consequences (Aven 2016, 2024; Renn et al. 2022). Ports exemplify such systems: they operate as interdependent sociotechnical networks where decision making and control are distributed among multiple actors. Under uncertainty, coordination mechanisms become essential for maintaining stable control and preventing risk amplification. Studies in risk analysis have demonstrated that coordination failures, whether in information sharing, crisis response, or communication, can significantly influence systemic outcomes (Ji and Fan 2025; Kim et al. 2022; Liu et al. 2025). These findings indicate that formal control structures alone are insufficient; dependable coordination mechanisms and the organizational arrangements that sustain them are equally important for maintaining accountability, resilience, and effective control across organizational boundaries.

Traditional risk‐analysis approaches have emphasized component reliability, probabilistic assessment, intraorganizational compliance, and safety controls (Aven 2016; N. Leveson 2012). However, contemporary risk science increasingly recognizes that systemic risks emerge through interconnected governance structures, uncertainty, and interdependencies across sociotechnical systems (Aven 2024; Renn et al. 2022). As a result, traditional approaches may provide limited visibility into the role of interorganizational coordination in analyzing systemic risk within fragmented operational environments.

Recent studies highlight that many safety issues in port systems emerge not from isolated failures, but from fragmented control, delayed feedback, and mismatched responsibilities among organizations (Bairami‐Khankandi et al. 2025; Milch and Laumann 2016; Nagi and Kersten 2022). Although prior studies have examined interorganizational risk management (IRM) in ports (Meléndez and Goerlandt 2025a, 2025b), they have largely treated coordination descriptively rather than operationalizing it as an analyzable component of systemic control structures. This limitation creates an important gap in understanding how coordination is both enacted through practical mechanisms and sustained by the organizational arrangements required to maintain dependable control across organizational boundaries.

System‐theoretic models, such as the Systems‐Theoretic Accident Model and Processes (STAMP) conceptualize safety as a problem of maintaining adequate control across interconnected sociotechnical systems, rather than preventing isolated component failures (Leveson 2004, 2011b). Building on this perspective, Meléndez and Goerlandt (2025a) developed a STAMP‐based Port Risk Control Structure (PRCS) that maps interorganizational control relationships, feedback loops, and responsibilities among port stakeholders. The PRCS provides the structural foundation for analyzing distributed control within complex port systems but does not explicitly address how coordination mechanisms are enacted and sustained across organizational boundaries.

This study addresses this gap by developing a systems‐based analysis approach that integrates the IRM‐CT with the PRCS. Within the proposed approach, coordination is conceptualized through adaptors, defined as observable organizational elements that connect distributed actors within interorganizational control systems. The IRM‐CT provides the taxonomy used to identify and classify these adaptors as either operational or enabling. Operational adaptors describe how coordination is enacted through mechanisms such as plans and rules, roles, routines, proximity, and objects and representations. Enabling adaptors describe the structural, procedural, and relational arrangements that sustain these mechanisms across organizational boundaries over time (Cherns 1976; Okhuysen and Bechky 2009; Reiman and Oedewald 2007).

The proposed approach embeds adaptor configurations within the PRCS to examine how coordination contributes to distributed control and feedback. It further distinguishes between three structural coordination states (“*Supported*,” “*Fragile*,” and “*Orphan*”) and evaluates adaptor configurations against three system value conditions: accountability, predictability, and common understanding, derived from Coordination Theory (Okhuysen and Bechky 2009) and aligned with principles of risk governance (ISO 2024) and resilience engineering (Hollnagel 2015). Together, these analytical stages provide an analytical basis for diagnosing coordination vulnerabilities and examining how coordination supports or constrains systemic risk governance.

Conceptually, this study contributes to risk analysis by integrating Coordination Theory, Sociotechnical Systems Theory, and Resilience Engineering within a unified systems‐based analysis approach. Rather than treating coordination as a contextual feature of organizational interaction, the proposed approach operationalizes it as an explicit analytical dimension of systemic risk analysis. Methodologically, the study introduces the IRM‐CT as a coordination taxonomy embedded within a STAMP‐based risk control structure, facilitating the systematic analysis of coordination across organizational boundaries. Empirically, it demonstrates a proof‐of‐concept application using the Halifax Port Authority (HPA) Port Information Guide (PIG; HPA 2024), illustrating how the proposed approach identifies coordination arrangements, produces an IRM Diagnosis, and evaluates their contribution to accountability, predictability, and common understanding through the system value analysis.

To the authors’ knowledge, this study is the first to develop a systems‐based analysis approach that explicitly integrates a coordination taxonomy within a STAMP‐based risk control structure to analyze interorganizational coordination.

The remainder of the paper is structured as follows. Section 2 describes the design science methodology and associated analytical procedures. Section 3 presents the proposed systems‐based analysis approach and its proof‐of‐concept application, detailing the IRM‐CT and its proof‐of‐concept application to the HPA context. Section 4 discusses the theoretical and practical implications of the findings and outlines avenues for future research. Section 5 concludes with a summary of contributions and final reflections.

## 2 Method

### 2.1 Research Approach

This study adopts a Design Science Research (DSR) methodology (Hevner et al. 2004), suitable for complex sociotechnical domains where the goal is both theoretical and practical advancement. DSR integrates problem relevance and research rigor through iterative design, evaluation, and refinement cycles (Opdenakker and Cuijpers 2025; Pastor et al. 2024).

The primary artifact developed in this study is the IRM‐CT, which serves as the analytical foundation of a systems‐based analysis approach for examining interorganizational coordination within STAMP‐based PRCS developed by Meléndez and Goerlandt (2025a). While the PRCS represents the control relationships, responsibilities, and feedback mechanisms within port systems, the IRM‐CT provides concepts for analyzing how coordination is enacted and sustained across these control structures. The taxonomy distinguishes between “*operational adaptors*,” which enact coordination in practice, and “*enabling adaptors*,” which provide the structural, procedural, or relational arrangements that sustain these mechanisms over time.

The DSR approach emphasizes four guiding principles: (i) problem relevance, by addressing persistent interorganizational coordination challenges in port systems; (ii) research rigor, by grounding the taxonomy in empirical and theoretical inputs; (iii) iterative refinement, through successive development stages; and (iv) communication, by reporting contributions to both academic and practitioner audiences.

**FIGURE 1:**
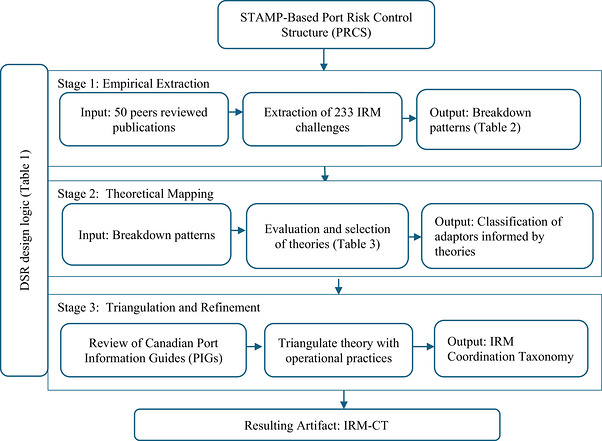
Methodological process for developing the IRM‐CT.

These principles are operationalized through three interconnected stages: the empirical extraction of IRM challenges, the theoretical mapping and integration of relevant perspectives, and the triangulation and refinement of the taxonomy using port documentation. These three stages, summarized in Figure 1, represent the logical design flow that links the identification of coordination challenges to the construction of the IRM‐CT and the resulting systems‐based analysis approach. Together, these stages provide the empirical, theoretical, and practical foundations used to develop and refine the taxonomy.

Building on this foundation, the study subsequently develops a systems‐based analysis approach that integrates the taxonomy with the STAMP‐based PRCS to support the examination of interorganizational coordination within distributed control systems. Accordingly, the DSR process supports the development of both the IRM‐CT as the primary design artifact and the analysis approach through which the artifact is applied within a PRCS.

### 2.2 Stage 1: Empirical Extraction of IRM Challenges

Stage 1 of Figure 1 established the empirical foundation of the taxonomy using findings from a previous study on IRM challenges in port operations and comparable high‐risk sectors (Meléndez and Goerlandt 2025b). The detailed search strategy, article‐selection process, coding procedures, and thematic analysis are reported in Meléndez and Goerlandt (2025b). Through systematic coding of 50 peer‐reviewed publications, 233 coordination‐related challenges were identified and grouped into 14 thematic categories spanning strategic, operative, and adaptive layers of control. These challenge categories served as the empirical foundation for taxonomy development and subsequent theory selection.

In line with the PRCS developed by Meléndez and Goerlandt (2025a), *strategic control* defines objectives and constraints across organizations, *operative control* governs the coordination and execution of interdependent activities, and *adaptive control* covers monitoring, feedback, and learning that support system adjustment under changing conditions (Leveson 2011b).

Each of the 14 categories was further associated with one of four dominant breakdown patterns observed across fragmented port systems: *control misalignment, fragmented coordination, sociotechnical gaps, and lack of adaptive capacity*. These patterns represent recurring ways in which coordination fails across organizational boundaries and thus served as the empirical criteria for guiding theory selection in Stage 2. Table 1 summarizes the 14 categories, their alignment with the three control layers, and their dominant breakdown patterns.

**TABLE 1:** IRM challenges categorized by control layer and dominant breakdown pattern.

IRM challenge category	Control layer	Dominant pattern of breakdown
Procedure obsolescence	Strategic control	Control misalignment
Strategic objective divergence	Strategic control	Fragmented coordination
Mental model misjudgment	Strategic control	Sociotechnical gap
Conflicting safety directives	Strategic control	Fragmented coordination
Authority and role design flaws	Operative control	Control misalignment
Operational decision gaps	Operative control	Fragmented coordination
Task execution gaps	Operative control	Control misalignment
Messaging disruptions	Operative control	Fragmented coordination
Schedule coordination failures	Operative control	Fragmented coordination
Resource constraints	Operative control	Lack of adaptive capacity
Technical system incompatibility	Adaptive control	Sociotechnical gap
External system disturbances	Adaptive control	Lack of adaptive capacity
Feedback loop disruptions	Adaptive control	Control misalignment
Perception gaps in system monitoring	Adaptive control	Sociotechnical gap

Grounding the IRM‐CT in empirically derived challenge categories ensured that it would address concrete interorganizational coordination challenges rather than relying solely on theoretical abstraction. The resulting problem structure served as a design brief for Stage 2, where complementary theoretical perspectives capable of addressing each breakdown pattern were identified, evaluated, and integrated.

### 2.3 Stage 2: Theoretical Mapping

Stage 2 translated the empirical findings into the theoretical foundation of the IRM‐CT and the systems‐based analysis approach (see Figure 1). Each breakdown pattern revealed a conceptual requirement that the taxonomy would need to address: control misalignment required explicit modeling of responsibilities, feedback, and constraints across system levels; fragmented coordination called for understanding how distributed actors align interdependent actions; sociotechnical gaps indicated the need to capture the interaction between organizational and technical arrangements; and lack of adaptive capacity emphasized the importance of responsiveness and learning under variability.

On this basis, four complementary theoretical perspectives were selected: the STAMP (Leveson 2004, 2011b), Coordination Theory (Malone and Crowston 1994), Sociotechnical Systems (STS) Theory (Cherns 1976), and Resilience Engineering (Hollnagel 2015). Each perspective contributes a distinct design function within the IRM‐CT and the resulting analysis approach. STAMP provides the control‐structure scaffolding necessary to represent interorganizational relationships; Coordination Theory defines the observable mechanisms through which work dependencies are managed; STS Theory clarifies how organizational arrangements sustain those mechanisms; and Resilience Engineering introduces value conditions for assessing dependable performance. Table 2 shows how each theory corresponds to the empirical patterns derived in Stage 1.

**TABLE 2:** Theoretical perspectives evaluated against selection criteria derived from IRM breakdown patterns.

Selection criterion (linked to breakdown pattern)	STAMP	Coordination Theory	STS Theory	Resilience Engineering
**Control structure framing** (control misalignment)	**Core**: Models control loops, responsibilities, and feedback explicitly	**Partial**: Coordination roles assumed, not structured in layers	**Partial**: Focus on system design implications, not control modeling	**Not emphasized**: Focus is on capacity, not structured control
**Interorganizational coordination mechanisms** (fragmented coordination)	**Partial**: Mentions interfaces between control layers	**Core**: Focus on coordinating actions across distributed actors	**Partial**: Considers interaction in sociotechnical design	**Partial**: Recognizes dynamic coordination needs under pressure
**Sociotechnical system alignment** (sociotechnical gaps)	**Partial**: Implicit through system levels and feedback, not detailed human/tech interaction	**Partial**: Considers coordination artifacts and routines	**Core**: Originates from studying human‐technology alignment	**Partial**: Adaptive capacity depends on human‐technology integration
**Adaptive capacity/temporal responsiveness** (lack of adaptive capacity)	**Not emphasized**: Focus on control layers.	**Partial**: Acknowledges timing and evolving coordination demands	**Partial**: Typically, static system design	**Core**: Anticipate, monitor, respond, and learn to improve future performance

*Note 1*: Each theory was selected for its core strength in one conceptual criterion. None alone is sufficient to construct a comprehensive taxonomy of IRM adaptors, hence their integration. The designations of “core,” “partial,” and “not emphasized” are based on how central each criterion is in the foundational literature of the theory. A classification was deemed “core” if the theory explicitly and systematically addresses the criterion, “partial” if it acknowledges or indirectly touches upon it, and “not emphasized” if it omits or minimally engages with the concept.

The IRM‐CT does not treat these theoretical perspectives as independent analytical layers. Rather, each theory addresses a specific coordination‐related limitation identified in Stage 1. STAMP provides the control‐structure foundation for representing distributed control and feedback relationships. Coordination Theory identifies the mechanisms through which coordination is enacted across organizational boundaries. Sociotechnical Systems Theory explains the organizational arrangements that sustain these mechanisms over time. Resilience Engineering provides the system value conditions used to assess coordination under uncertainty and variability. Together, these perspectives form the theoretical foundation of the IRM‐CT and the systems‐based analysis approach, enabling the systematic examination of interorganizational coordination as an explicit component of distributed control structures.

#### 2.3.1 Systems‐Theoretic Accident Model and Processes

STAMP reconceptualizes safety as a problem of inadequate control rather than component failure, emphasizing hierarchical structures, system constraints, and feedback loops (Leveson 2004, 2011b). As shown in Table 2, STAMP is core for control structure framing, corresponding to the empirical pattern of control misalignment. It explicitly models how responsibilities, constraints, and feedback are distributed across system levels, providing a basis for diagnosing where coordination‐related control deficiencies emerge across organizational boundaries.

In this study, the IRM‐CT and the associated analysis approach draw on nine STAMP control functions that define the structure of the PRCS (Meléndez and Goerlandt 2025a): *Controller, Control Logic, Control Action, Actuator, Controlled Process, Sensor, Feedback, Disturbance, and Constraint*. The PRCS was previously developed by Meléndez and Goerlandt (2025a) using STAMP/STPA principles for modeling control structures, responsibilities, feedback relationships, and constraints within complex sociotechnical systems (Leveson 2011b; Leveson and Thomas 2018). Detailed guidance on the development of control structures is provided in the STPA Handbook (Leveson and Thomas 2018). In the present study, the PRCS serves as the structural representation upon which the IRM‐CT is applied.

These nine control functions serve as the systemic reference structure used to embed operational and enabling adaptors within the PRCS. Operational adaptors such as rules, roles, or routines can be positioned relative to controllers, control logic, or control actions, while enabling adaptors can be linked to how constraints and responsibilities are embedded across organizational boundaries.

By explicitly situating coordination mechanisms within these nine control functions, the IRM‐CT and the associated analysis approach move beyond descriptive classification and enable the systematic examination of coordination within a STAMP‐based control structure. This systemic positioning clarifies how adaptors contribute to maintaining control, enforcing constraints, and sustaining feedback loops across fragmented organizational boundaries.

#### 2.3.2 Coordination Theory

Coordination Theory explains how distributed actors manage dependencies in interdependent work systems (Malone and Crowston 1994). Dependencies can occur between tasks, resources, and actors, and coordination mechanisms provide the means to manage them. Okhuysen and Bechky (2009) synthesized this literature into five generic categories of coordination mechanisms: *plans and rules, objects and representations, roles, routines, and proximity*. These categories form the conceptual basis of the taxonomy's operational adaptors, which represent the observable coordination mechanisms through which interorganizational coordination is enacted in port operations.

As shown in Table 2, Coordination Theory is core for interorganizational coordination mechanisms, directly addressing the breakdown pattern of fragmented coordination identified in Stage 1. Ports often involve multiple independent organizations whose activities must be synchronized; when mechanisms such as communication protocols, checklists, or designated roles are absent, duplicated, or inconsistently applied, coordination breaks down.

By linking these mechanisms to PRCS functions, for example, aligning rules and routines with control logic, or situating objects and representations within feedback loops, the taxonomy enables systematic coding and analysis of operational adaptors. This ensures that coordination is not only described in abstract terms but explicitly situated within a system‐theoretic model of safety, allowing operational adaptors to be analyzed in relation to control relationships, feedback loops, and interorganizational control structures.

#### 2.3.3 Sociotechnical Systems Theory

Sociotechnical systems (STS) theory emphasizes the principle of joint optimization, where social and technical subsystems must be designed together to achieve effective organizational performance (Baxter and Sommerville 2011; Cherns 1976; Clegg 2000). Rather than treating coordination as purely procedural or technological, STS highlights how structures, procedures, and relational arrangements condition the functioning of coordination mechanisms in practice.

In the taxonomy, these arrangements are defined as enabling adaptors that provide the structural, procedural, and relational conditions necessary for operational adaptors to operate consistently across organizational boundaries. Examples include formal organizational arrangements, memoranda of understanding (MoUs), and joint standard operating procedures (SOPs) that provide the organizational “scaffolding” necessary for coordination mechanisms to remain robust. This perspective is also consistent with research on interorganizational networks, which emphasizes the role of institutionalized arrangements in supporting coordination, managing interorganizational dependencies, and sustaining collective action across organizational networks (Clauss and Ritala 2023).

As shown in Table 2, STS Theory is core for sociotechnical system alignment, directly addressing the empirical pattern of sociotechnical gaps identified in Stage 1. Such gaps emerge when coordination mechanisms are not adequately embedded in organizational arrangements, for instance, when a communication protocol exists but is not reinforced by shared procedures or relational trust between organizations.

Linking STS to the PRCS enables the analysis of not only where coordination occurs in the control structure but also how it is sustained. By making enabling adaptors explicit, the taxonomy extends prior PRCS work (Meléndez and Goerlandt 2025a), which had largely mapped control functions without clarifying the enabling organizational arrangements required to sustain coordination mechanisms and support dependable control across organizational boundaries.

#### 2.3.4 Resilience Engineering

Resilience Engineering focuses on how complex systems sustain performance under conditions of variability by anticipating, monitoring, responding, and learning (Hollnagel 2012, 2015). It shifts the focus from preventing failures to ensuring that organizations can adapt to both expected and unexpected changes. Within the taxonomy, Resilience Engineering provides the theoretical rationale for assessing whether coordination arrangements support adaptive capacity under variability and uncertainty. This assessment is operationalized through the system value conditions of accountability, predictability, and common understanding identified by Okhuysen and Bechky (2009). Together, these conditions provide the basis for assessing coordination is likely to remain dependable in practice.

As shown in Table 2, Resilience Engineering is core for adaptive capacity, directly addressing the empirical pattern of lack of adaptive capacity identified in Stage 1. When accountability is unclear, decision making becomes slow or inconsistent; when predictability is limited, thresholds and triggers for action are absent; and when common understanding is lacking, organizations fail to form a shared situational picture. These vulnerabilities have been repeatedly observed in fragmented port operations, where coordination mechanisms exist but their effectiveness depends heavily on operator compliance or resource availability (Meléndez and Goerlandt 2025b).

By linking resilience concepts to the PRCS, the taxonomy adds an analytical dimension. Operational adaptors such as drills, dashboards, or routines can be examined not only for their formal design but for whether they genuinely support accountability, predictability, and common understanding across organizations. Enabling adaptors such as interagency agreements or formal coordination arrangements can then be assessed for how they enable these conditions to emerge. To ground these conditions more firmly in Resilience Engineering, Table 3 shows how each value condition aligns with Hollnagel's (2015) four resilience potentials (anticipate, monitor, respond, and learn). This mapping clarifies the pathways through which coordination mechanisms and arrangements contribute to dependable system performance.

**TABLE 3:** Mapping of system value conditions to Hollnagel's (2015) resilience potentials.

Resilience potential	Accountability	Predictability	Common understanding
**Anticipate**	Assigned owners for risk scanning and scenarios.	Codified triggers/scenarios make future actions foreseeable.	Shared mental models of emerging risks.
**Monitor**	Named monitoring roles and audit trails.	Defined indicators/thresholds and timing.	Standard phraseology/shared status displays for situation awareness.
**Respond**	Clear decision rights and authority to act.	Preauthorized procedures and escalation paths enable timely action.	Read backs/liaison roles coordinate intent across actors.
**Learn**	Ownership for debriefs and implementing changes.	SOP/version updates and change control capture lessons.	Dissemination of lessons aligns interpretations across organizations.

*Note 2*: These are primary pathways; in practice, the three value conditions can reinforce multiple resilience potentials.

Together, these theories explain how coordination is enacted through operational adaptors, sustained through enabling adaptors, embedded within distributed control structures, and examined through system value conditions that support the diagnosis of coordination vulnerabilities.

### 2.4 Stage 3: Triangulation and Refinement

Stage 3 refined and evaluated the taxonomy through empirical triangulation (see Figure 1). Six Canadian PIGs (HPA 2024; MPA 2023; Port of Vancouver 2025; Prince Rupert Port Authority 2024; PRPA 2025; Toronto Port Authority 2000) were examined to determine whether operational and enabling adaptor categories could be identified in real‐world documentation. PIGs codify sanctioned interorganizational practices for vessel traffic management, emergency response, and cargo handling, representing work‐as‐imagined (WaI) under regulatory conditions (Hollnagel 2014; Wears and Hollnagel 2017)

This stage served two main purposes. First, supported taxonomy refinement by testing whether operational and enabling adaptors categories could be consistently identified within real‐world documentation, ensuring that the taxonomy could be operationalized beyond abstract theorizing, a necessary aspect of reality‐based safety science (Rae et al. 2020). Second, it the evaluation of the taxonomy through a form of convergent validity (Sadeghi and Goerlandt 2023), cross‐checking whether the taxonomy built from literature and theoretical integration aligned with practices formally recognized in Canadian port operations.

Ambiguities led to refinements in inclusion and exclusion criteria, for example, distinguishing when a joint role description constitutes an operational adaptor versus an enabling adaptor. Mechanisms that appeared redundant were re‐evaluated as potential coordination clutter rather than necessary safeguards. Overlaps between operational and enabling adaptors were resolved by treating them as level‐dependent: operational adaptors describe observable coordination mechanisms, while enabling adaptors represent the structural or relational conditions that sustain them, without introducing additional adaptor categories.

Importantly, all proposed adaptor categories were identified at least once across the six PIGs reviewed, and no additional adaptor categories emerged during triangulation. While the analysis led to refinements in category definitions and classification criteria, the empirical review provided support for the practical applicability and empirical coverage of the taxonomy.

The six PIGs used during Stage 3 supported taxonomy refinement and evaluation, whereas the HPA application presented in Section 3 serves as the final proof‐of‐concept demonstration of the resulting systems‐based analysis approach.

### 2.5 Development of the IRM‐CT Analysis Approach

The systems‐based analysis approach emerged during the iterative design and refinement cycles of the DSR process. As the IRM‐CT was developed and examined against empirical port documentation, it became apparent that a systematic procedure was needed to relate operational and enabling adaptors to the control relationships represented in the PRCS. The resulting approach integrates the taxonomy with the PRCS to support the analysis of interorganizational coordination within distributed control structures.

While the development of the IRM‐CT provides a structured classification of coordination mechanisms and supporting arrangements, the contribution of this study extends beyond the taxonomy itself. The study proposes a systems‐based analysis approach for analyzing interorganizational coordination within the PRCS by integrating the analytical concepts of the IRM‐CT with the STAMP‐based representation of control relationships developed by Meléndez and Goerlandt (2025a).

The rationale for this integration is that the PRCS provides a representation of organizational actors, control relationships, feedback channels, and responsibilities, while the IRM‐CT provides concepts for analyzing how coordination is enacted and sustained within these relationships. The combined use of both artifacts enables a systematic examination of coordination structures that would not be possible through either artifact independently. Whereas the PRCS makes visible how control and feedback are distributed across organizational boundaries, the IRM‐CT provides concepts for identifying the mechanisms through which coordination occurs and the arrangements that support these mechanisms over time.

The analysis approach begins by examining the PRCS to identify coordination mechanisms embedded within control and feedback relationships among organizational actors. These mechanisms are classified as operational adaptors using the categories of the IRM‐CT. The analysis then examines the structural, procedural, and relational arrangements that support the identified coordination mechanisms, which are conceptualized as enabling adaptors. By analyzing operational and enabling adaptors together, the approach enables the diagnosis of coordination states as “*Supported*,” “*Fragile*,” or “*Orphan*.”

The resulting adaptor configurations are subsequently examined in relation to the three system value conditions of accountability, predictability, and common understanding. This assessment provides insight into the extent to which coordination arrangements contribute to dependable system control across organizational boundaries. The proposed approach therefore focuses not only on identifying coordination mechanisms but also on examining the conditions that sustain them. It further highlights the vulnerabilities that may arise when enabling arrangements are absent, misaligned, or insufficiently developed.

The outcome of the analysis is a qualitative diagnosis of coordination structures within the PRCS. Rather than performing a quantitative risk assessment, the approach supports the identification of coordination vulnerabilities, revealing areas where control may be compromised by insufficient enabling arrangements, fragmented responsibilities, or inadequate feedback. In this way, the integration of the IRM‐CT and the PRCS provides a systematic means for analyzing how coordination is structured, sustained, and embedded within complex interorganizational systems.

## 3 Results

### 3.1 IRM Coordination Taxonomy: Overview

The principal outcome of this study is the development of an IRM‐CT aligned with the PRCS. The taxonomy provides a structured way to analyze coordination through adaptors, defined as the observable organizational elements that allow interdependent actors to align actions within distributed risk‐management systems.

Adaptors may be material or symbolic, including protocols, roles, dashboards, notification routines, or infrastructures. Hutchins (1995), such elements mediate distributed cognition, while Star and Griesemer (1989) describe them as boundary objects that bridge perspectives between groups. To capture both the enactment and sustainment of coordination, the taxonomy distinguishes between *operational adaptors* (which describe how coordination is enacted in practice) and *enabling adaptors* (which denote the organizational arrangements that sustain this enactment across boundaries). The distinction is grounded in coordination theory (Malone and Crowston 1994; Okhuysen and Bechky 2009) and sociotechnical systems theory (Cherns 1976; Clegg 2000). The IRM‐CT builds on the empirical analysis of 233 IRM challenges developed by Meléndez and Goerlandt (2025b), which demonstrated that many failures arose not from the absence of coordination mechanisms but from insufficient or missing arrangements needed to sustain their consistent use (see Section 2.2).

The interaction between operational and enabling adaptors may result in different coordination configurations, which are later assessed through the application process described in Section 3.2.1.

**FIGURE 2:**
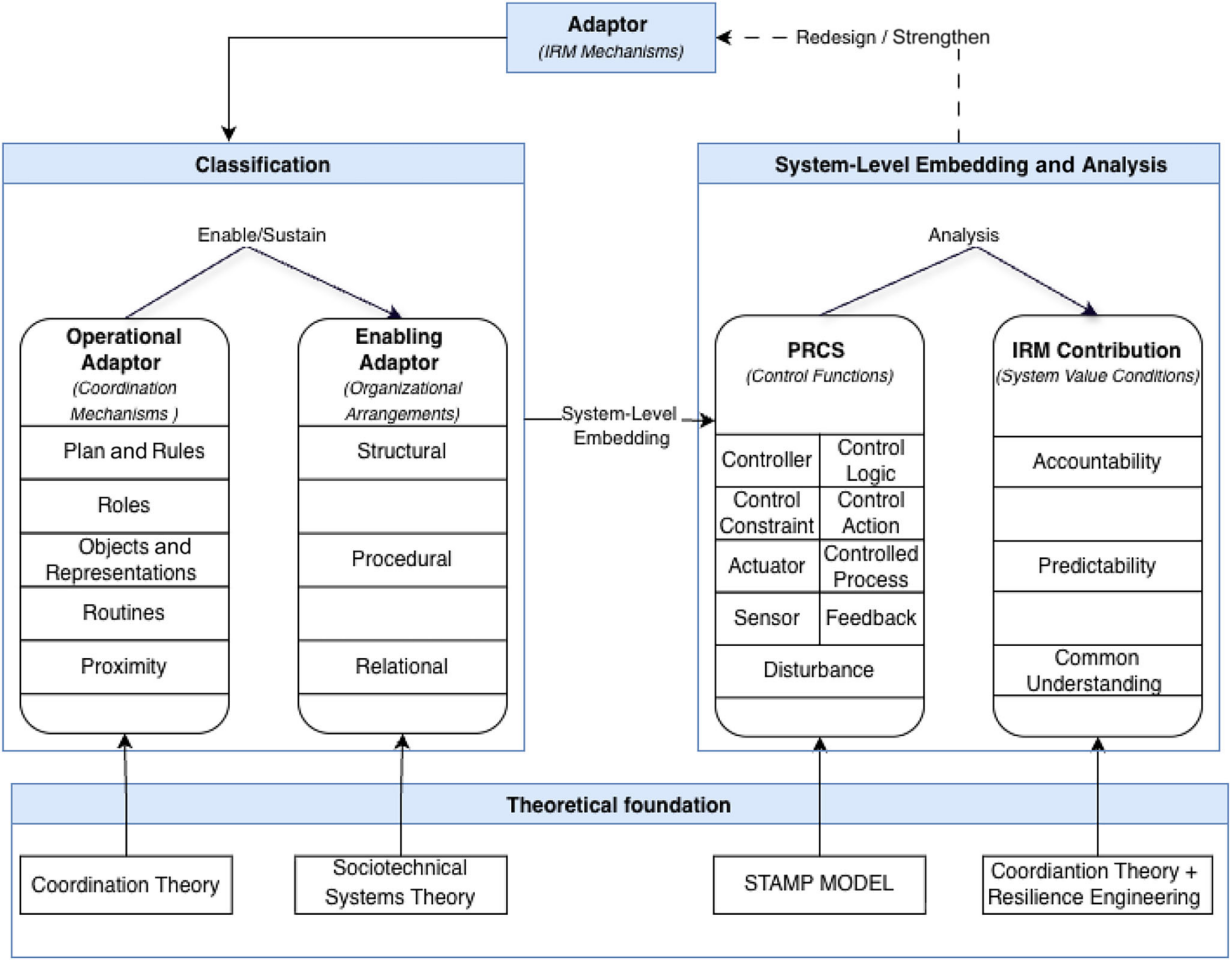
Conceptual representation of the IRM coordination taxonomy.

Figure 2 presents the conceptual representation of the IRM‐CT. Within the systems‐based analysis approach, the taxonomy is used to classify coordination arrangements into operational and enabling adaptors and to examine the relationships between them. First**, classification** identifies adaptors and distinguishes them into operational and enabling categories. During this stage, linkage verification examines whether operational adaptors are supported by enabling arrangements and whether enabling adaptors are connected to observable coordination mechanisms, allowing the identification of “*Supported*,” “*Fragile*,” and “*Orphan*” coordination states. Second, **system‐level embedding** situates adaptors within the PRCS using STAMP control functions as defined by Leveson (2004, 2011b). Embedding adaptors in systemic control loops links coordination practices to their role in maintaining system‐wide risk control. Third, **analysis** assesses adaptor configurations against three system value conditions: *accountability, predictability, and common understanding*. Following Okhuysen and Bechky (2009) and resilience engineering principles (Hollnagel 2015), these conditions capture the outcomes that effective coordination must generate in interorganizational risk‐management systems. Adaptors that fail to deliver these conditions risk becoming clutter, understood as activities or artifacts maintained in the name of safety but with limited contribution to coordination, control, or systemic safety outcomes (Hutchinson et al. 2022; Rae et al. 2018).

The taxonomy incorporates an iterative element: analytical results can inform the redesign and strengthening of adaptors, consistent with resilience engineering principles of monitoring, adaptation, and learning (similar to the continuous‐improvement logic of the Deming PDCA cycle). The taxonomy is therefore not only a classification scheme but an analytical tool that extends the PRCS by making coordination explicit as a system element. The following subsections elaborate each stage of the taxonomy, detailing the subcategories of operational and enabling adaptors, their embedding within STAMP control functions, and their evaluation through system value conditions.

#### 3.1.1 Operational Adaptors

Operational adaptors constitute the primary coordination mechanisms analyzed within the taxonomy and capture how coordination is enacted in practice. They represent the observable mechanisms through which interdependent actors align their actions. These adaptors correspond directly to the five categories of coordination mechanisms (Malone and Crowston 1994; Okhuysen and Bechky 2009), which conceptualizes how interdependent work is organized and synchronized.

Within the taxonomy, operational adaptors are grouped into five categories. *Plans and rules* prescribe responsibilities, sequences, or procedures (e.g., predeparture checklists). *Roles* designate authority and accountability for decisions (e.g., Port Security Officer). *Routines* are recurrent processes such as weekly berth‐allocation meetings or safety drills. *Objects and representations* are artifacts that make information visible and shareable, such as dashboards or logs. *Proximity* encompasses real‐time alignment achieved through colocation or communication technologies that create a shared operational space among distributed actors, such as the use of VHF channels that create a shared operational space for pilots, vessels, and authorities.

These categories make explicit the functional modes through which coordination is enacted in daily port operations. Table 4 summarizes their definitions, guiding questions, and examples, supporting systematic identification and classification of operational adaptors within the conceptual structure of the taxonomy illustrated in Figure 2. Their contribution to coordination, however, depends on the presence of enabling adaptors, which provide the authority, codification, and trust required to sustain them across organizational boundaries.

**TABLE 4:** Operational adaptors: Definitions, guiding questions, and examples.

Operational adaptor category	Definition	Guiding question	Example (port context)
**Plans and rules**	Prescriptive operational adaptors that assign responsibilities, sequence tasks, or impose procedures.	Does this adaptor prescribe who does what and in what order?	Predeparture safety checklist for vessels.
**Objects and representations**	Operational adaptors that make information visible and interpretable across actors.	Does this adaptor provide a shared representation for coordination?	Electronic port traffic monitoring dashboard.
**Roles**	Operational adaptors that designate formal authority and accountability for decision making.	Does this adaptor specify who has authority or accountability for decisions?	Port Security Officer acting as the primary point of contact for all port security matters
**Routines**	Operational adaptors that institutionalize recurrent processes, structuring repeated activities or reporting cycles.	Does this adaptor formalize repeated activities or reporting?	Weekly berth‐allocation coordination meeting.
**Proximity**	Operational adaptors that enable real‐time coordination through colocation, visibility, or direct communication.	Does this adaptor rely on physical/digital proximity for coordination?	VHF Channel 12 for pilot, vessel–port communications.

#### 3.1.2 Enabling Adaptors

Enabling adaptors are the organizational arrangements that ensure operational adaptors do not remain unsupported or disconnected from the conditions required to sustain them across organizational boundaries. Drawing on STS theory and the empirical findings from Stage 1, enabling adaptors recognize that coordination mechanisms must be embedded within organizational structures and relationships to function consistently across organizational boundaries (Cherns 1976; Clegg 2000).

Three types are distinguished. “*Structural adaptors*” are enduring arrangements, such as offices, mandates, or governance bodies, that embed authority and oversight within the system. “*Procedural adaptors*” are codified pathways, such as SOPs, MoUs, or approval frameworks, that formalize and standardize coordination practices across organizational actors. “*Relational adaptors*” are trust‐based arrangements, such as liaison roles, informal networks, or joint working groups, that complement and reinforce formal structures and procedures, sustaining coordination particularly in situations where flexibility, mutual understanding, and rapid communication are required.

The mandatory use of VHF Channel 12 in Halifax Harbour illustrates their interaction. The channel operates as an operational adaptor within the proximity category, enabling real‐time coordination and shared situational awareness. Its reliable use, however, depends on structural adaptors (the port authority mandate), procedural adaptors (standardized message formats and sequences), and relational adaptors (mutual trust that messages will be transmitted, interpreted, and acted upon). Without these enabling adaptors, the VHF channel risks becoming a technical artifact that exists in regulation but does not guarantee dependable coordination.

As discussed in Section 2.4, classification is level dependent. An element that functions as an operational adaptor at one level of the PRCS may serve as an enabling adaptor at another level if its primary role is to sustain coordination mechanisms rather than directly enact them. For example, a regulatory mandate may function as an operational adaptor at a higher governance level because it directly structures and coordinates the actions of subordinate organizations. The same mandate may function as an enabling adaptor when examined from the perspective of local port operations, where its role is to provide the authority and institutional basis that sustains operational coordination mechanisms implemented by port actors.

Table 5 provides definitions, guiding questions, and illustrative examples for each type of enabling adaptor. As shown in Figure 2, these guiding questions support consistent classification by anchoring judgments in systematic reasoning rather than subjective interpretation.

**TABLE 5:** Adaptors as enabling arrangements: Definitions and guiding questions.

Adaptor type	Definition	Guiding question	Example (port context)
**Structural**	Enduring organizational arrangements (e.g., offices, mandates, governance bodies) that do not carry out coordination themselves but guarantee that coordination mechanisms are applied consistently by embedding authority and responsibility into the system.	Does this adaptor establish or mandate authority that guarantees the consistent use of coordination mechanisms across organizations?	The Harbour Master's office requiring submission of a predeparture checklist before vessel clearance.
**Procedural**	Codified arrangements (e.g., SOPs, MoUs, standardized approval pathways) that ensure coordination mechanisms are enacted uniformly across organizations, protecting them from ad‐hoc or inconsistent application.	Does this adaptor provide formalized documentation or codification that sustains the consistent use of coordination mechanisms in practice?	A memorandum of understanding (MoU) specifying information exchange between port authority and customs during cargo inspections.
**Relational**	Trust‐based or liaison arrangements (e.g., informal networks, boundary‐spanning roles) that do not replace mechanisms but sustain their interpretation and enactment across organizational boundaries, especially where formal structures or procedures are insufficient.	Does this adaptor rely on trust, liaison roles, or interpersonal networks to sustain coordination mechanisms across organizational boundaries?	Informal cooperation between tug operators and pilots that ensures last‐minute adjustments to departure timing are respected.

The conceptualization of enabling adaptors is also consistent with broader perspectives on network governance, which emphasize the role of institutionalized arrangements in supporting coordination, managing interorganizational tensions, and sustaining collective value creation across organizational boundaries (Clauss and Ritala 2023). From this perspective, structural, procedural, and relational adaptors can be interpreted as institutionalized governance mechanisms that support the continued functioning of coordination processes within complex interorganizational systems.

#### 3.1.3 Embedding in STAMP Control Functions

Classifying adaptors as operational or enabling provides insight into how coordination is enacted and sustained but does not explain how these arrangements contribute to systemic control. As illustrated in Figure 2, to evaluate their systemic role, adaptors must also be embedded in the broader PRCS. This embedding uses the STAMP control functions already introduced in Section 2, which conceptualize safety not as component reliability but as the ability to maintain adequate control of system behavior (Leveson 2004, 2011b). In this taxonomy, the PRCS (Meléndez and Goerlandt 2025a) provides the structural backbone that situates adaptors within control and feedback loops, thereby linking coordination practices to systemic risk management.

The control functions employed in this analysis include controllers, control logic, control actions, actuators, controlled processes, sensors, feedback, constraints, and disturbances (Table 6). By embedding adaptors into these functions, the taxonomy makes explicit how coordination contributes to the maintenance of system‐level control across organizational boundaries rather than treating it as an isolated activity. For example, an operational adaptor such as a checklist may represent control logic, while an enabling adaptor such as a mandate from the Harbour Master's office provides the structural authority that guarantees its use. Similarly, communication protocols can serve as feedback mechanisms, sustained by procedural adaptors such as standardized phraseology requirements.

**TABLE 6:** STAMP control functions and their role in classifying adaptors.

STAMP control function	Definition	Guiding question (applied to adaptors)
**Controller**	Organizational actor(s) responsible for issuing control actions	Does this adaptor assign or support a decision‐making role?
**Control logic**	How the controller perceives the system and decides actions	Does this adaptor define or codify decision rules?
**Control constraints**	Safety rules and boundaries that control actions must respect	Does this adaptor impose or sustain limits on actions?
**Control actions**	Commands or decisions issued to influence the system	Does this adaptor issue or enable control actions?
**Actuators**	Mechanisms implementing control actions	Does this adaptor identify who executes the action?
**Controlled process**	Operational system being managed	Does this adaptor regulate or sustain a port operation?
**Sensors**	Data collection mechanisms informing controllers	Does this adaptor enable monitoring or data collection?
**Feedback**	Information returning to the controller about system state	Does this adaptor facilitate the communication, interpretation, or use of information that informs subsequent control actions?
**Disturbances**	External uncontrollable events impacting the system	Does this adaptor anticipate, buffer, or respond to disruptions?

*Note 2*: These control functions provide the basis for embedding operational and enabling adaptors within the PRCS and analyzing their contribution to system‐level control and feedback.

This embedding step also addresses a recurrent limitation observed in fragmented port systems, where coordination mechanisms often exist in documentation but are disconnected from systemic feedback loops (Hutchinson et al. 2022; Milch and Laumann 2016). By explicitly associating adaptors with STAMP functions, the taxonomy prevents such mechanisms from being treated as symbolic or peripheral or disconnected from meaningful control and feedback processes. Instead, it situates them within the PRCS as integral components of distributed control. Table 6 summarizes the control functions used and provides guiding questions that help determine how each adaptor contributes to maintaining control and feedback within the PRCS.

#### 3.1.4 System Value Conditions

Even when properly embedded within control structures, adaptors may not generate dependable coordination. The taxonomy therefore introduces three system value conditions: “*accountability*,” “*predictability*,” and “*common understanding*.” These conditions, articulated by Okhuysen and Bechky (2009) and aligned with resilience engineering principles (Hollnagel 2015), capture the qualities that coordination arrangements should exhibit to support dependable coordination across organizational boundaries.

“*Accountability*” refers to clear responsibility and authority for actions, approvals, and outcomes. “*Predictability*” concerns the reliability of timing, sequencing, and expected results, enabling actors to anticipate one another's actions. “*Common understanding*” reflects the degree to which information, procedures, and signals are interpreted consistently across organizations. Table 7 summarizes these conditions, offering definitions, guiding questions, and port‐related examples.

**TABLE 7:** System value conditions: Definitions and guiding questions.

Condition	Definition	Guiding question	Example (port context)
Accountability	The extent to which responsibility and authority for coordination are clearly established and recognized across organizational boundaries.	Does this adaptor clarify who is responsible for specific actions, approvals, or outcomes in the coordination process?	A Declaration of Security (DoS) protocol that specifies whether the Harbour Master, terminal operator, or vessel master is accountable for approving security measures before operations.
Predictability	The ability of the system to ensure reliable timing, sequencing, and outcomes of interdependent tasks, enabling actors to anticipate one another's actions.	Does this adaptor ensure that timing, sequencing, or outcomes of interdependent tasks are reliable and consistent?	An Estimated Time of Arrival (ETA) reporting process that synchronizes vessel movements and aligns operational schedules across multiple port stakeholders.
Common understanding	The degree to which information, procedures, and signals are interpreted consistently across organizations, ensuring shared meaning in coordination.	Does this adaptor support alignment of interpretations and shared meaning across different organizations?	Standardized phraseology used on VHF communication channels that ensures pilots, tug operators, and vessel crews interpret messages in the same way.

Together, the system value conditions provide an analytical lens that complements classification and embedding. They move the taxonomy from describing how adaptors are structured and embedded within the PRCS toward assessing the extent to which adaptor configurations support accountability, predictability, and common understanding across organizational boundaries. These assessments form the basis of the “*Strong*,” “*Moderate*,” and “*Weak*” ratings introduced in Section 3.2.3.

### 3.2 Application of the IRM Coordination Taxonomy

**FIGURE 3:**
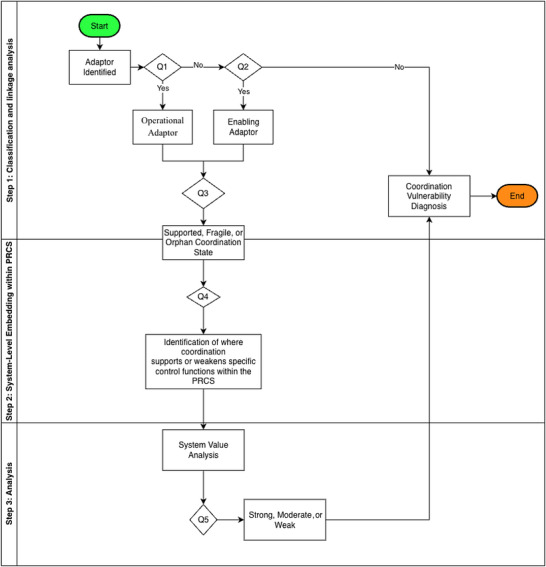
Systems‐based analysis approach for diagnosing coordination vulnerabilities using the IRM‐CT and PRCS.

As illustrated in Figure 3, the IRM‐CT is applied through a three‐stage analytical process: classification and linkage analysis, system‐level embedding, and system value analysis. The proposed approach does not evaluate quantitative performance but offers a structured process for interpreting how coordination artifacts operate across interorganizational boundaries and for identifying where control and feedback relationships may be vulnerable or absent.

The application of the IRM‐CT assumes the existence of a system control structure model that represents the relevant actors, control relationships, and feedback mechanisms within the domain of interest. In this study, the taxonomy is applied using the PRCS developed by Meléndez and Goerlandt (2025a). In other domains, equivalent control structures may be developed using established STAMP‐based modeling approaches prior to applying the taxonomy.

The conceptual logic for these analytical stages was introduced in Section 3.1, see Figure 2. The following subsections describe how each stage supports systematic analysis, from the identification and classification of adaptors, through their embedding within the PRCS, to the assessment of accountability, predictability, and common understanding. Table 8 summarizes the taxonomy's dimensions, categories, and guiding analytical questions used throughout this process.

**TABLE 8:** Analytical workflow for applying the IRM‐CT within the PRCS.

Step	Taxonomy dimension	Categories	Referenced table	Diagnostic question
**1. Classification and linkage verification**	**Operational adaptors**	Plans and rules; roles; routines; objects and representations; proximity	Table 4	**Q1**: Does this adaptor directly facilitate coordination in practice by prescribing tasks, defining authority, structuring routines, or enabling proximity?
	**Enabling adaptors**	Structural; procedural; relational	Table 5	**Q2**: Does this adaptor provide authority, codification, or trust that sustains coordination across organizational boundaries?
	**Cross‐mapping between operational and enabling adaptors**	Linked pairs of adaptors	—	**Q3**: Is the operational adaptor supported by one or more enabling adaptors, and are enabling adaptors connected to an observable coordination mechanism? Classify the relationship as “*Supported*,” “*Fragile*,” or “*Orphan*.”
**2. Systemic embedding**	**Integration within the PRCS**	Controllers; control logic; control actions; actuators; controlled processes; sensors; feedback; constraints; disturbances	Table 6	**Q4**: Where does this adaptor (or linked pair) fit within the STAMP control functions?
**3. System value analysis**	**System value conditions**	Accountability; predictability; common understanding	Table 7	**Q5**: To what extent does the adaptor pair contribute to accountability, predictability, and common understanding? Classify as “*Strong*,” “*Moderate*,” or “*Weak*.”

Table 8 and Figure 3 summarize the systems‐based analysis approach developed in this study. Figure 3 provides a visual overview of the analytical workflow, while Table 8 details the associated diagnostic questions and classification criteria. The analysis proceeds sequentially. First, operational and enabling adaptors are identified and classified (Q1–Q2), followed by linkage verification to determine “*Supported*,” “*Fragile*,” and “*Orphan*” coordination states (Q3). These coordination states are then embedded within the PRCS to identify where coordination influence specific control functions (Q4). Finally, the embedded coordination states are evaluated against the system value conditions of accountability, predictability, and common understanding (Q5), resulting in a System Value Assessment of “*Strong*,” “*Moderate*,” or “*Weak*.”

Before classification, analysts must define the level of analysis within the PRCS. Because adaptor functions are level‐dependent and context‐dependent, the same element may operate as an enabling adaptor at one control level and as an operational adaptor at another. Classification should therefore be performed relative to the focal coordination process and the corresponding control relationships under examination. Defining the level of analysis establishes the analytical boundary for the assessment and helps ensure consistent adaptor classification.

#### 3.2.1 Step 1: Classification and Linkage Analysis

The first step classifies each identified adaptor according to its functional role in coordination. “*Operational adaptors*” describe how coordination is enacted in practice, through plans, routines, representations, or proximity, while “*enabling adaptors*” represent the organizational arrangements that sustain these practices, such as mandates, procedures, or trust networks. As represented in Table 8, the classification proceeds through two diagnostic questions: Q1 identifies operational adaptors that directly facilitate coordination, and Q2 identifies enabling adaptors that provide the conditions for their consistent enactment.

Following classification, the relationships between adaptors are examined to determine whether operational and enabling adaptors are appropriately connected. Specifically, the analysis verifies whether each operational adaptor is “*Supported*” by at least one enabling adaptor and whether each enabling adaptor sustains one or more operational adaptors. Absent or insufficient linkages indicate coordination vulnerabilities. A “*Fragile*” operational adaptor exists when a coordination mechanism is present but lacks sufficient enabling support, whereas an “*Orphan*” enabling adaptor exists when supporting arrangements are present without an observable operational mechanism to enact them. This verification step ensures that coordination is analyzed as an interdependent structure rather than as a collection of isolated adaptors.

The diagnostic states shown in Table 9 describe the structural relationship between operational and enabling adaptors and provide an initial structural diagnosis of coordination arrangements. These diagnostic outcomes form the basis for the next stage of the analysis, in which adaptor pairs are embedded within the PRCS by mapping them to the relevant STAMP control functions. This systemic embedding enables subsequent evaluation of how adaptor configurations contribute to accountability, predictability, and common understanding through the system value analysis.

**TABLE 9:** Diagnostic coordination states within the IRM‐CT.

Diagnostic state	Operational adaptor	Enabling adaptor	Interpretation
“*Supported*”	Present	Present	Coordination is adequately supported by enabling arrangements.
“*Fragile*”	Present	Absent or insufficient	Coordination exists but is vulnerable due to limited enabling support.
“*Orphan*”	Absent	Present	Supporting arrangements exist without an identifiable coordination mechanism.

The classification process assumes that the relevant PRCS level has already been defined. Operational and enabling adaptor classifications are assigned relative to that level of analysis.

#### 3.2.2 Step 2: Systemic Embedding in the PRCS

The second step, system‐level embedding, situates the adaptor configurations identified in Step 1 within the PRCS (Meléndez and Goerlandt 2025a) using the STAMP control functions introduced in Section 2. As summarized in Table 8, this stage is guided by Q4, which examines where the adaptor (or linked pair) fits within the STAMP control functions. Mapping follows Leveson's (2004, 2011b) model, applying functions such as controller, control logic, control action, actuator, controlled process, sensor, feedback, constraint, and disturbance.

Embedding adaptors configurations within these functions connects coordination practices to their systemic purpose within the PRCS. Analysts can trace how operational and enabling adaptors contribute to system control and identify where coordination mechanisms are present, disconnected, insufficiently sustained, or absent within control and feedback loops. This stage provides the basis for identifying opportunities to strengthen specific control functions, such as improving feedback processes, clarifying control logic, reinforcing control constraints, or addressing missing enabling arrangements.

#### 3.2.3 Step 3: System Value Analysis

The final step evaluates whether adaptors configurations against the three system value conditions introduced in Section 3.1.4. As summarized in Table 8, this stage is guided by Q5, which examines the extent to which an adaptor pair contributes to accountability, predictability, and common understanding. Building on the structural diagnosis and systemic embedding conducted in the previous stages, analysts assess whether the configuration of operational and enabling adaptors sustains these values within the PRCS. The result is a system value assessment that classifies each adaptor pair as “*Strong*,” “*Moderate*,” or “*Weak*.” Table 10 summarizes the assessment criteria.

**TABLE 10:** Assessment criteria for system value analysis.

Performance rating	Assessment criteria	Interpretation
“*Strong*”	Accountability, predictability, and common understanding are all clearly evident.	The adaptor pair consistently supports effective coordination across organizational boundaries.
“*Moderate*”	One or more value conditions are only partially achieved, but the adaptor pair still contributes meaningfully to coordination.	Coordination is generally effective but may be vulnerable to variability, resource constraints, or changing operational conditions.
“*Weak*”	One or more value conditions are largely absent, significantly limiting coordination effectiveness.	The adaptor pair provides insufficient support for reliable coordination and may require redesign or reinforcement.

This system value assessment complements the structural diagnosis presented in Stage 1. Whereas the “*Supported*,” “*Fragile*,” and “*Orphan*” states describe the structural relationship between operational and enabling adaptors, the “*Strong*,” “*Moderate*,” and “*Weak*” ratings reflect the extent to which those relationships contribute to accountability, predictability, and common understanding within the PRCS.

### 3.3 Proof‐of‐Concept: Applying the IRM‐CT to the Halifax Port Authority Port Information Guide

To demonstrate the applicability and internal consistency of the proposed taxonomy, a convergent validity test is conducted as a proof‐of‐concept using the HPA PIG (HPA 2024). These documents specify sanctioned procedures and interagency roles and therefore represent “WaI”, that is, the prescribed way in which coordination and risk management are expected to occur according to formal rules and documentation. This concept contrasts with “work‐as‐done” (WaD), which refers to how work and coordination are actually carried out in practice, often adapting to real‐world variability (Rae and Provan 2019). In this study, the PIG is used as a formal, WaI reference to demonstrate how the IRM‐CT can be applied to analyze and diagnose interorganizational coordination mechanisms and their supporting arrangements. The HPA application serves as a proof‐of‐concept demonstration of the taxonomy and should not be interpreted as a comprehensive assessment of coordination within the port.

Adaptors are extracted as verbatim clauses (≤ 25 words) from HPA (2024) representing coordination elements relevant to safety, security, and operational coordination. Extraction followed a close reading and qualitative content analysis approach (Baker and Mcenery 2017) allowing the identification of coordination mechanisms described in the text.

As detailed in Table 8, each adaptor is analyzed using the IRM‐CT through three analytical stages. First, classification and linkage analysis identifies operational and enabling adaptors and examines their relationships, resulting in an IRM diagnosis of “*Supported*,” “*Fragile*,” or “*Orphan*.” Second, systemic embedding situates adaptor configurations within the PRCS using the corresponding STAMP control function (e.g., controller, control logic, control action, feedback, constraint, and sensor). Third, system value analysis evaluates the extent to which adaptor configurations support accountability, predictability, and common understanding, resulting in a System Value Assessment of “*Strong*,” “*Moderate*,” or “*Weak*.”

This analytical process, described in Section 3.2 and conceptually represented in Figure 2, enables each adaptor configuration to be systematically traced through the PRCS, connecting documented coordination practices to their role in sustaining systemic control and feedback across organizational boundaries. Table 11 presents five illustrative adaptors from the HPA PIG (HPA 2024) selected to represent the five operational adaptor categories outlined in Section 3.1. These examples are illustrative rather than exhaustive and are intended to demonstrate the application of the taxonomy across different forms of coordination. Together, they demonstrate how the taxonomy can be applied to diagnose coordination structures and assess their contribution to accountability, predictability, and common understanding.

**TABLE 11:** Application of the IRM‐CT to selected HPA adaptors.

Adaptor (source)	Operational adaptor	Enabling adaptors	IRM diagnostic	PRCS functions	System value conditions assessment	
**VHF channel communications** (HPA PIG, 14.8, 2025): “A continuous monitoring watch must be maintained on VHF Channels 12 and 16.”	Proximity: Real‐time coordination via radio communication between vessels, pilots, and HPA.	**Structural** (HPA mandate for channel use); **Procedural** (standardized message format and sequence); **Relational** (mutual trust in response and clarity).	**Supported**	**Feedback**, **Sensor**: Channels transmit and receive updates and detect anomalies in traffic flow.	**Accountability**: Supported (vessels and pilots responsible for continuous watch). **Predictability**: Conditional (depends on equipment reliability and discipline). **Common understanding**: Conditional (risk of misinterpretation across crews and languages).	**Moderate**
**Declaration of Security (DoS)** (HPA PIG, 10.2, 2025): “The purpose of a Declaration of Security is to ensure agreement is reached between the vessel and the port facility.”	**Plans and rules**: Formal agreement defining security responsibilities between vessel and facility.	**Structural** (MFSO mandate under MTSR); **Procedural** (DoS completion requirements); **Relational** (cooperation during signing).	**Supported**	**Control constraint**, **Control logic**, **Control action**: Defines security boundaries and formal authorizations.	**Accountability**: Supported (MFSO responsible for DoS completion). **Predictability**: supported (triggered under codified MARSEC conditions). **Common understanding**: supported (shared expectations through agreement).	**Strong**
**Coordination Meeting for Nonconventional Vessels** (HPA PIG, 8.2, 2025): “Agent initiates communication after passage plan approval by HPA in consultation with APA and agents.”	**Routines**: Recurrent approval and coordination process for special movements.	**Structural** (HPA approval authority); **Procedural** (plan approval requirement); **Relational** (interagency consultation among APA, HPA, agents).	**Supported**	**Controller**, **Control logic**, **Control action**, **Feedback**: Establish decision and information flow for vessel movements.	**Accountability**: Supported (HPA approves, APA consults, agent initiates). **Predictability**: supported (rule ensures routine activation for special cases). **Common understanding**: Conditional (challenged as actors increase).	**Moderate**
**Enforcement Officer inspections** (HPA PIG,15.3, 2026): “An HPA Enforcement Officer may board any vessel and conduct inspections…”	**Roles**: Designated authority for law enforcement and compliance oversight.	**Structural** (Canada Marine Act, 108 mandate); **Procedural** (inspection protocols); **Relational** (authority recognized by Masters and crew).	**Supported**	**Controller**, **Control constraint**, **Control action**, **Sensor**, **Feedback**: Exercise authority, collect evidence, and inform HPA.	**Accountability**: Supported (clear legal mandate). **Predictability**: Supported (standardized enforcement powers). **Common understanding**: Supported (role universally recognized).	**Strong**
**International Ship Security Certificate (ISSC)** (HPA PIG, 5, 2025): “The following documents must be available on board at all times, including the ISSC.”	**Objects and representations**: Physical certificate demonstrating compliance with ISPS Code.	**Structural** (ISPS Code requirement); **Procedural** (PIG inspection mandate); **Relational** (trust in certificate as evidence of compliance).	**Supported**	**Sensor**, **Control constraint**, **Feedback**: Signals security status and assures compliance during inspection.	**Accountability**: Supported (vessel operator responsible for validity). **Predictability**: Supported (global standardization). **Common understanding**: Supported (universal recognition across ports).	**Strong**

*Note 3*: Technical abbreviations are provided in Appendix A.

Across the five examples, all coordination mechanisms were classified as “*Supported*” under the IRM Diagnosis, indicating the presence of identifiable enabling arrangements. However, the System Value Assessment revealed variation in the extent to which adaptor configurations contributed to accountability, predictability, and common understanding. While regulatory‐ and compliance‐oriented mechanisms generally achieved “*Strong*” assessments, several operational adaptors reported in Table 11, particularly those relying on information exchange and coordination across organizational boundaries, were more frequently assessed as “*Moderate*” due to dependencies on interpretation, information quality, and operational variability.

The “*Moderate*” assessments observed for VHF communications and coordination meetings illustrate how structurally “*Supported*” coordination mechanisms may still be vulnerable to operational variability. In practice, such vulnerabilities may increase the likelihood of communication delays, inconsistent interpretation of information, or reduced coordination during nonroutine operations. For example, a missed or misunderstood VHF communication could delay the transmission of navigational instructions, increasing the potential for operational disruption, near misses, or other safety‐critical events. Similarly, coordination delays during nonconventional vessel movements may reduce the ability of multiple actors to maintain a shared situational picture during complex operations.

This proof‐of‐concept demonstrates the taxonomy's capacity to systematically trace how coordination mechanisms and their enabling structures are embedded within control loops and aligned with system value conditions. The application shows that the proposed approach can be operationalized using existing regulatory documentation, producing structured analytical outputs that distinguish between the structural relationships captured by the IRM Diagnosis and the contribution of adaptor configurations to accountability, predictability, and common understanding captured by the System Value Assessment. The analysis further illustrates how the proposed approach can identify opportunities for improvement by highlighting where coordination may benefit from additional enabling arrangements, strengthened control and feedback functions, or improvements in accountability, predictability, and common understanding. While the results are limited to a WaI context, they provide an initial demonstration of the analytical logic of the proposed approach and establish a foundation for future empirical applications in WaD settings.

## 4 Discussion

### 4.1 Reflection on Findings

The proof‐of‐concept analysis demonstrates the analytical potential of the IRM‐CT for making interorganizational coordination structures visible within formal port governance. Applying the taxonomy to the PIG showed how documented coordination practices, conceptualized as adaptors, function as observable components of systemic control. By classifying adaptors, embedding them in the PRCS functions, and evaluating them against system value conditions, the IRM‐CT reveals the interdependencies that sustain or constrain coordination across organizational boundaries. Beyond generating diagnostic insights, it shows how coordination arrangements are structured, sustained, and embedded within interorganizational control systems.

The analysis also shows that the classification of adaptors is relational rather than fixed. At the port level, the Harbour Master's role in Table 9 functions as an enabling adaptor because it embeds authority and oversight sustaining multiple operational adaptors, such as communication routines, reporting protocols, and coordination meetings. At the national PRCS level, however, the same role becomes an operational adaptor, itself sustained by higher level enabling adaptors such as the Canada Marine Act and guidance from Transport Canada. Thus, what counts as an enabling or operational adaptor depends on the control layer under consideration, illustrating that coordination is nested across governance levels rather than confined to a single organizational tier.

From this multilevel perspective, local coordination arrangements cannot be assessed in isolation. Their effectiveness depends on the alignment and adequacy of enabling adaptors at national and international levels. The IRM‐CT therefore helps reveal where coordination vulnerabilities may emerge when higher level structures diverge, become insufficient, or fail to reinforce operational practices, allowing analysts to diagnose vertical misalignment that may compromise system‐wide safety and risk control.

The results also highlight variation in the contribution of adaptor configurations to the system value conditions. The VHF channel communication requirement, for instance, is diagnosed as “*Supported*” but receives a “*Moderate*” System Value Assessment (Table 11). Although formally mandated and structured, its reliability depends on disciplined operator behavior, technical functionality, and consistent interpretation across multilingual crews. The role of Enforcement Officers is grounded in statutory authority, yet practical effectiveness may still be constrained by resources or inspection capacity. Even globally standardized mechanisms such as the ISSC can become vulnerable if outdated or falsified, thereby undermining predictability and common understanding. These examples illustrate how the IRM‐CT distinguishes between the structural configuration of coordination arrangements and the conditions that influence their performance in practice.

More broadly, the proof‐of‐concept highlights the limits of analyzing only WaI sources such as PIGs. These documents codify sanctioned procedures and thus tend to portray coordination as stable and coherent. In practice, discrepancies arise between WaI and WaD, where adaptations, shortcuts, and resource constraints reshape coordination (Hutchinson et al. 2022, 2024) The taxonomy helps identify where such gaps are likely, but empirical validation through observations and stakeholder interviews remains necessary to determine whether mechanisms that appear robust on paper function effectively in daily operations. Triangulating documentation analysis with WaD evidence is therefore a key direction for future work.

Overall, the proof‐of‐concept validates the analytical logic of the IRM‐CT. It shows that the taxonomy can operationalize complex coordination concepts within a system‐theoretic model, revealing both structural alignment and potential vulnerabilities. By combining classification, systemic embedding, and value‐based analysis, the taxonomy provides a structured yet flexible approach for analyzing coordination dependencies and guiding targeted interventions in port risk control systems.

### 4.2 Theoretical Contribution

Conceptually, this study advances systemic risk analysis by extending STAMP‐based approaches to include an explicit analytical perspective on IRM. It enhances the PRCS by introducing the IRM‐CT as a taxonomy within a systems‐based analysis approach for classifying and analyzing coordination mechanisms across organizational boundaries. While STAMP‐based methods such as STPA focus on causal and control interactions within systems (Leveson and Thomas 2018), they provide limited insight into the coordination mechanisms and organizational arrangements that sustain interorganizational control in practice. The IRM‐CT addresses this gap by embedding coordination elements, termed adaptors, into STAMP control functions and evaluating their contribution to system value conditions.

In doing so, the study links Coordination Theory, Sociotechnical Systems theory, and Resilience Engineering within a unified analytical model. Coordination is not treated merely as context, but as a formal analytical dimension of safety and risk control structures. This responds to calls in risk and safety science to better capture systemic interactions and governance arrangements in complex sociotechnical systems.

Unlike previous studies that primarily identified or categorized interorganizational risk‐management challenges (Meléndez and Goerlandt 2025b), the IRM‐CT provides a structured analytical mechanism for examining how coordination is enacted, sustained, and embedded within systemic control structures. This shifts coordination from a descriptive characteristic of interorganizational interaction to an explicit object of analysis within systemic risk governance. In contrast to traditional risk‐analysis approaches that primarily examine hazards, probabilities, or organizational controls, the IRM‐CT explicitly examines how coordination itself contributes to maintaining systemic control across organizational boundaries.

The taxonomy also contributes to the IRM literature by offering a structured analytical tool for assessing coordination in interorganizational settings. Prior work has largely catalogued IRM challenges, such as communication breakdowns or feedback delays, without providing operational tools to diagnose how these manifests in concrete systems (Meléndez and Goerlandt 2025a; Milch and Laumann 2016). The IRM‐CT bridges this gap by enabling analysts to trace how coordination mechanisms are embedded in control loops, sustained by enabling structures, and linked to the system value conditions of accountability, predictability, and common understanding.

The IRC‐CT may also contribute to advancing understanding of how complex systems drift into failure (Dekker 2011; Rasmussen 1997; Scott 2000), by revealing where coordination mechanisms, though formally present, rely on conditions that may degrade over time, such as continuous operator compliance, informal workarounds, or limited resource capacity. By linking adaptors and systemic values conditions, the IRM‐CT provides a structured way to detect early signs of coordination degradation before they escalate into operational disruptions. While further empirical work is required to confirm this diagnostic capacity, the proposed approach provides an initial structure for detecting latent vulnerabilities that often remain obscured in routine interorganizational operations (Reason 2015).

The taxonomy may also support the exploration of coordination‐failure scenarios and their potential propagation across interorganizational control structures. For example, a VHF communication routine may be classified as a “*Supported*” coordination mechanism but receive only a “*Moderate*” System Value Assessment because its contribution to accountability, predictability, and common understanding depends on disciplined operator behavior and reliable communication practices. Under adverse conditions, delays or misinterpretations in communication could prevent timely transmission of navigational instructions, increasing the likelihood of operational disruptions, near misses, or other safety‐critical events. Similarly, “*Orphan*” enabling adaptors may indicate the presence of plans, mandates, or organizational arrangements that are formally established but not effectively enacted through operational coordination mechanisms, creating vulnerabilities during emergency or nonroutine situations.

The taxonomy may also contribute to anticipatory forms of analysis by helping organizations identify coordination vulnerabilities before they manifest as operational failures. Recent work on foresight and preparedness in port systems highlights the importance of governance mechanisms that enable organizations to anticipate and respond to emerging disruptions (Attanasiko et al. 2024). By identifying “*Fragile*” coordination arrangements, “*Orphan*” enabling adaptors, and limitations in system value conditions, the proposed approach provides an analytical basis for examining how governance structures may enable or constrain future preparedness and adaptive capacity.

The IRM‐CT responds to critiques of safety clutter, defined as the accumulation of safety‐related activities, documents, or controls that consume resources but do not meaningfully contribute to operational safety (Provan et al. 2017; Rae et al. 2018). By distinguishing adaptors that genuinely support the PRCS functions from those that lack systemic connections, the taxonomy helps identify where coordination exists largely in documentation. Situations in which enabling adaptors are not linked to operational adaptors, or vice versa, may indicate symbolic compliance rather than functional coordination. This resonates with the distinction between “safety work” and “the safety of work” (Provan et al. 2019): the taxonomy helps assess whether coordination artifacts that look robust on paper actually sustain accountability, predictability, and common understanding in practice.

To the authors’ knowledge, this study represents the first attempt to operationalize interorganizational coordination as an explicit analytical component of a STAMP‐based risk control structure. By integrating Coordination Theory, Sociotechnical Systems theory, Resilience Engineering, and empirical evidence from interorganizational risk‐management challenges, the IRM‐CT extends existing approaches by providing a structured method for identifying, classifying, embedding, and assessing coordination arrangements within systemic risk governance.

### 4.3 Practical Implications

While further validation is required before recommending the practical application of the proposed work, the IRM‐CT provides ports and regulators with a structured analytical approach for assessing the contribution of coordination mechanisms to systemic safety and risk management. By classifying adaptors and analyzing them through the PRCS functions, and value conditions, the IRM‐CT moves beyond compliance checks to reveal how coordination is sustained within control and feedback loops.

In practice, the proposed approach can support internal safety reviews, regulatory audits, and interagency coordination exercises. Communication routines or security documentation that appear procedurally robust on paper may require additional monitoring, training, or resourcing if their effectiveness depends on conditions such as disciplined operator behavior or adequate inspection capacity. Conversely, adaptor configurations that consistently reinforce accountability, predictability, and common understanding can be identified as important contributors to systemic resilience.

For example, a port authority could apply the IRM‐CT to analyze emergency‐response coordination arrangements. The assessment may reveal that formal emergency‐response procedures exist as operational adaptors but are only partially sustained by enabling adaptors such as clearly assigned coordination authorities, codified communication procedures, or established interorganizational relationships. Such findings could guide targeted improvements by strengthening the organizational arrangements needed to sustain reliable coordination during emergency operations.

The taxonomy also enables cross‐organizational dialog by providing a shared analytical language. Port authorities, shipping agents, regulators, and other stakeholders can use it in existing collaborative settings (such as safety committee meetings, emergency preparedness roundtables, or joint incident reviews) to discuss whether adaptors are genuinely contributing to coordination or risk drifting into redundancy, across organizational borders. This diagnostic framing creates a basis for prioritizing resources, strengthening vulnerable coordination links, and preventing the accumulation of practices that consume resources without meaningfully contributing to coordination.

The taxonomy does not quantify risk, likelihood, or consequence severity. Consequently, not all “*Fragile*” coordination arrangements should be assumed to present the same level of concern. In practice, organizations may combine the taxonomy with expert judgment, scenario analysis, or existing risk‐assessment processes to evaluate the criticality of identified vulnerabilities and prioritize mitigation efforts. For example, a communication‐related vulnerability may be considered more critical when associated with vessel traffic management than when linked to a routine administrative process. In this way, the proposed approach can complement broader risk‐assessment activities without replacing them.

The IRM‐CT may have broader applicability to other coordination‐intensive sociotechnical systems. Preliminary exploratory research applying the taxonomy within the aviation domain identified analogous operational and enabling adaptor structures in aviation safety‐management documentation, suggesting that the underlying coordination concepts extend beyond maritime operations (Meléndez and Goerlandt 2026a). Furthermore, an initial transferability assessment presented by Meléndez and Goerlandt (2026b) demonstrated how the IRM‐CT can be applied to the analysis of maritime accident investigations, highlighting its potential to support the identification and characterization of coordination arrangements in different safety‐critical contexts. While additional empirical evaluation is required, these studies provide preliminary evidence of the IRM‐CT's applicability beyond ports and support its potential use for examining coordination strengths and vulnerabilities across diverse interorganizational systems.

Finally, by embedding adaptors within the PRCS, the taxonomy gives regulators and policymakers a clearer understanding of how local coordination depends on higher level enabling structures at national and international levels. This multilayered perspective can inform the design of policies and oversight mechanisms, ensuring that structural mandates (e.g., legislation or international codes) effectively cascade into local adaptors that sustain control, feedback, and collective safety performance across the port system.

### 4.4 Future Research

Future research should build on this study by empirically validating the across multiple organizational settings. Because the present evaluation relies primarily on formal PIG documentation, it is limited to WaI. Additional research is therefore needed to examine how adaptor configurations function in daily operations, how coordination degrades or adapts under variability, and how the IRM Diagnosis and System Value Assessment align with observed coordination practices and outcomes. Future validation efforts should include stakeholder‐based assessments, inter‐rater reliability testing, comparative cross‐port studies, and WaD investigations to evaluate the robustness and practical utility of the proposed approach.

A practical near‐term avenue is the systematic analysis of PIGs across multiple port authorities. As shown in Meléndez and Goerlandt (2025a), PIGs codify sanctioned procedures and coordination roles, providing a consistent WaI baseline. Applying the proposed systems‐based analysis approach to a broader set of PIGs could support the development of a large‐scale diagnostic of interorganizational coordination and help identify where empirical follow‐up is most needed. PIGs themselves may be interpreted as operational adaptors at a national PRCS level, supported by structural adaptors such as the Canada Marine Act. At the local port level, the same documents function as enabling adaptors that sustain operational coordination mechanisms, illustrating the level‐dependent nature of adaptor classification. Comparative studies could examine how this transition occurs across different regulatory contexts and what it reveals about coordination layering and vertical misalignment within the PRCS structures.

Methodologically, further work should test the inter‐rater reliability of the proposed systems‐based analysis approach and refine its diagnostic criteria to ensure consistent application, following an approach similar to that adopted by Laine et al. (2025). Establishing consistent classifications across evaluators represents an important prerequisite for assessing the transferability of the taxonomy beyond the contexts examined in this study. Such work would help determine whether operational and enabling adaptors, IRM diagnoses, and system value assessments can be applied consistently by different analysts.

Future research could also explore how the proposed systems‐based analysis approach relates to uncertainty and to emerging Safety‐II perspectives. In the present study, uncertainty is understood broadly as arising from variability in operational conditions, organizational interactions, and coordination processes, as well as from incomplete knowledge about system states, responsibilities, dependencies, and the actions of other organizational actors (Aven 2022, 2023). While the proposed approach does not explicitly assess uncertainty, its application may help identify coordination arrangements that are vulnerable to such uncertainties within interorganizational control structures. Future work could further examine how different adaptor configurations influence the management of uncertainty across organizational boundaries and whether the proposed approach can support their analysis. It could also explore how these relationships connect with emerging discussions in risk science and resilience engineering.

Finally, future studies should evaluate the transferability of the proposed systems‐based analysis approach across different interorganizational sociotechnical systems. Such applications would help determine whether adaptor configurations, enabling arrangements, and system value conditions exhibit similar patterns across domains and operational contexts. Beyond ports, sectors such as aviation, mining, healthcare, and energy provide promising contexts for evaluating the proposed approach and examining how coordination mechanisms contribute to systemic control and risk governance. Examining these questions would provide further evidence regarding the scope, limitations, and generalizability of the proposed systems‐based analysis approach beyond its initial development within port operations.

## 5 Conclusion

This study developed a systems‐based analysis approach that integrates the IRM‐CT with the STAMP‐based PRCS to examine how coordination mechanisms are enacted, sustained, and evaluated within complex interorganizational sociotechnical systems. The proposed approach operationalizes interorganizational coordination as an explicit analytical dimension of systemic risk governance by combining taxonomy‐based classification, system‐level embedding, and system value analysis.

The proof‐of‐concept application using the HPA PIG demonstrated the analytical logic of the proposed approach. It showed how documented coordination mechanisms could be systematically classified as operational and enabling adaptors, embedded within the PRCS control functions, and evaluated against the system value conditions of accountability, predictability, and common understanding. Together, these analytical stages distinguish between the structural characteristics of coordination arrangements (IRM Diagnosis) and their contribution to dependable coordination (system value analysis). This provides an analytical basis for identifying coordination vulnerabilities and opportunities to strengthen systemic risk governance.

Conceptually, the study contributes to risk analysis by integrating Coordination Theory, Sociotechnical Systems Theory, and Resilience Engineering within a unified analytical approach for examining interorganizational coordination. Rather than treating coordination as contextual background, the proposed approach makes it an explicit object of analysis within the STAMP‐based PRCS. In doing so, it complements existing systems‐theoretic approaches by providing a structured means of examining how coordination arrangements support, constrain, or degrade distributed control across organizational boundaries.

Practically, the proposed systems‐based analysis approach could provide regulators, port authorities, and other stakeholders with a structured analytical framework for examining coordination arrangements within existing governance systems. Rather than replacing established risk‐assessment methods, it complements them by identifying coordination vulnerabilities, examining whether coordination arrangements are adequately supported, and highlighting opportunities to strengthen enabling structures and reduce safety clutter. Although further empirical validation is required before broad practical application can be recommended, the proposed approach also appears applicable to other coordination‐intensive sociotechnical systems, including aviation, healthcare, mining, and energy.

The principal contribution of this study lies not only in the development of the IRM‐CT but also in the proposal of a systems‐based analysis approach that integrates the taxonomy with the PRCS. Together, these contributions extend systems‐based risk analysis beyond representing control structures toward the systematic examination of interorganizational coordination, enabling analysts to diagnose coordination vulnerabilities and explore opportunities to strengthen systemic risk governance.

Future research should evaluate the transferability, reliability, and practical applicability of the proposed approach across multiple domains and operational contexts. Particular attention should be given to integrating WaD evidence, evaluating inter‐rater reliability, and examining how adaptor configurations evolve under operational variability and uncertainty. Comparative cross‐sector studies could further assess how coordination influences resilience, uncertainty management, and systemic drift. Preliminary applications in aviation and maritime accident investigation suggest that the underlying coordination concepts may extend beyond port operations, providing an initial basis for broader empirical evaluation.

Ultimately, the proposed systems‐based analysis approach contributes to ongoing developments in systemic risk analysis by providing a structured means of identifying, embedding, and evaluating coordination arrangements within distributed control structures. By making interorganizational coordination an explicit analytical dimension of systemic risk governance, the approach offers a foundation for examining how coordination contributes to the robustness, adaptability, and governance of complex sociotechnical systems.

## Conflicts of Interest

The authors declare no conflicts of interest.

## 1

**Table:** Appendix A List of abbreviations

Abbreviation	Full term
APA	Atlantic Pilotage Authority
DoS	Declaration of Security
HPA	Halifax Port Authority
HPA PIG	Halifax Port Authority Port Information Guide
IRM	Interorganizational risk management
ISSC	International Ship Security Certificate
MARSEC	Maritime Security Level
MFSO	Marine Facility Security Officer
MoU	Memorandum of understanding
MCTS	Marine Communications and Traffic Services
PRCS	Port Risk Control Structure
PIG	Port Information Guide
SOP	Standard operating procedure
STAMP	Systems‐Theoretic Accident Model and Processes
VHF	Very high frequency (radio communication)
WaD	Work‐as‐done
WaI	Work‐as‐imagined
